# Pulmonary-Renal Syndrome with Negative ANCAs and Anti-GBM Antibody

**DOI:** 10.1155/2013/434531

**Published:** 2013-10-31

**Authors:** Hiroshi Yamaguchi, Atsuhisa Shirakami, Takashi Haku, Takashige Taoka, Yoshikazu Nakanishi, Toru Inai, Takanori Hirose

**Affiliations:** ^1^Department of Diabetology and Metabolic Medicine, Tokushima Prefectural Central Hospital, 1-10-3, Kuramoto, Tokushima 770-8539, Japan; ^2^Department of Internal Medicine, Tokushima Prefectural Central Hospital, 1-10-3, Kuramoto, Tokushima 770-8539, Japan; ^3^Department of Respiratory Medicine, Tokushima Prefectural Central Hospital, 1-10-3, Kuramoto, Tokushima 770-8539, Japan; ^4^Department of Urology, Tokushima Prefectural Central Hospital, 1-10-3, Kuramoto, Tokushima 770-8539, Japan; ^5^Department of Diagnostic Pathology, Tokushima Prefectural Central Hospital, 1-10-3, Kuramoto, Tokushima 770-8539, Japan

## Abstract

We report the case of a 76-year-old woman who was referred to our hospital for a gradually worsening cough and renal dysfunction. Although pneumonia was initially suspected, imaging findings of the lungs revealed diffuse alveolar hemorrhage at a later date. Renal failure developed and hemodiafiltration was performed on the 9th day. Rapidly progressive glomerulonephritis with crescent formation was diagnosed by renal biopsy. This case presentation has important clinical implications because uncategorizable pulmonary-renal syndrome (PRS) without the presence of ANCAs and anti-GBM antibody is extremely rare and has high rates of morbidity and mortality. No treatment has been established.

## 1. Introduction

Pulmonary renal-syndrome (PRS), characterized by a combination of diffuse alveolar hemorrhage (DAH) and rapidly progressive glomerulonephritis (RPGN), is caused by varied etiologies, including Goodpasture's syndrome, antineutrophil cytoplasmic antibody (ANCA)-associated small vessel vasculitis (ASVV), cryoglobulinemia, systemic lupus erythematosus, environmental factors, and certain drugs [1, 2]. ASSV, which can be caused by microscopic polyangiitis (MPA), Wegener's granulomatosis, and Churg-Strauss syndrome, accounts for approximately 70% of the cases of PRS. Antiproteinase-3 (anti-PR3, c-ANCA) and antimyeloperoxidase (anti-MPO, p-ANCA) antibodies, which have been reported to play a major role in the pathogenesis of ASSV, are detectable in 70–90% of cases and facilitate diagnosis. 

Our present patient could not be classified into known subgroups because all commercially available serologic studies were negative. Therefore, a renal biopsy was performed for diagnostic clues at a later date. As a result, PRS was diagnosed on the basis of clinical manifestations and pathological findings. 

## 2. Case Report

A 76-year-old Japanese woman presented to our hospital complaining of a worsening cough of 10-day duration. Her past medical history included hypertension since age 70. Her activities of daily living had been independent before admission. On admission, her vital signs were as follows: body temperature, 35.3°C; heart rate, 86 beats/min; SpO_2_, 96% (on room air); respiratory rate, 14 breaths/min; and blood pressure, 161/78 mmHg. No skin lesions or peripheral neuropathy were noted. 

**Figure 1:**
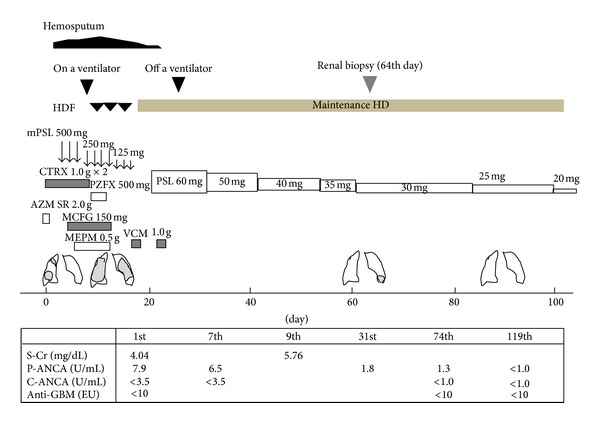
The patient's clinical course.

A laboratory examination revealed leukocytosis (white blood cell count [WBC]: 11,900/mm), normocytic normochromic anemia (Hb: 6.1 g/dL), elevation of C-reactive protein (CRP) level (6.1 mg/dL), and renal dysfunction (BUN: 42.6 mg/dL, Cr: 4.04 mg/dL). Urinalysis showed the following results: proteinuria 3+ (1.48 g/day) and hematuria 3+. The red cell counts in the urinary sediment were elevated (388 per high-power field). Serological tests for MPO-ANCA, PR3-ANCA, and anti-GBM antibodies were performed using enzyme-linked immunosorbent assay (ELISA). Antineutrophil cytoplasm antibodies against myeloperoxidase (MPO-ANCA) and antibodies against proteinase 3 (PR3-ANCA) and antiglomerular basement membrane (anti-GBM) antibodies were not identified, although these assays were performed several times during the clinical course (Figure 1). Testing for hepatitis B and C viruses, and antinuclear antibody titer was negative. The complement levels were normal.

**Figure 2:**
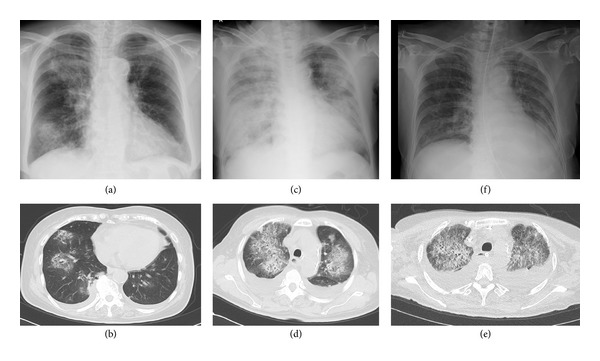
(a), (b) Chest X-ray and CT on admission showed bilateral central opacities, which are more confluent in the right upper and lower lung zone than elsewhere. There is slight cardiomegaly. (c) The radiograph obtained after intubation on the 10th day showed increased confluence of the opacities. The right hemidiaphragm is partially obscured. (d) Chest CT scan on the 10th day showed bilateral ground-glass opacities corresponding to the central and perihilar opacities and both small pleural effusions. (e) Chest CT scan on the 36th day showed progressed bilateral ground-glass opacities, confluent, and consolidated opacities, and a large quantity of pleural effusions in both lung fields. (f) Chest X-ray on the 92nd day showed the disappearance of infiltrative shadows in both lung fields.

Chest X-ray and thoracic computed tomography (CT) scan revealed patchy alveolar infiltrates throughout the upper and lower zones in both lungs (Figures 2(a) and 2(b)). 

An electrocardiogram showed normal sinus rhythm. Echocardiography revealed a normal ejection fraction of 60%, normal right-ventricular size, no evidence of a pericardial effusion, and no findings that were consistent with pulmonary edema. Abdominal ultrasound showed bilateral normal-sized kidneys with increased echogenicity. 

Pneumonia was suspected on admission and several antibiotics were initiated based on an increase in inflammatory reaction including WBC and CRP levels. However, as alveolar patchy infiltrates gradually expanded after admission (Figures 2(c), 2(d), and 2(e)) and she expectorated bloody sputum on the 3rd day, we evaluated these antibiotic drugs as ineffective and she was treated with a blood transfusion because of worsening of anemia.

To determine the causative organism, culture tests were performed for sputum culture five times and blood culture three times over the course of hospitalization; however, no bacterial species causing pneumonia were detected. Serological tests for *Cytomegalovirus, Mycoplasma,* and fungus were also negative. No infectious explanation for these imaging findings was found.

On the 10th day, as the oxygen saturation fell to 82% despite the administration of oxygen (10 liters per minute) by face mask, she was moved to the intensive care unit (ICU) and the trachea was intubated and mechanical ventilation was begun. Her renal function worsened with serum creatinine increasing steadily from 4.04 mg/dL on admission to a peak of 5.76 mg/dL on the 9th day. Rapidly progressive glomerulonephritis (RPGN) was diagnosed by urinary findings and progressive loss of renal function. As there had been no urine output on the 9th day, a hemodialysis catheter was placed, and hemodiafiltration (HDF) was begun. 

The findings of chest X-ray and chest CT scan indicated the more specific group of disorders known as diffuse alveolar hemorrhage accompanied by glomerulonephritis. Therefore, the patient was started on 500 mg of methylprednisolone intravenously daily for 3 days under the administration of antibiotics on the 9th day. After that, methylprednisolone was sequentially administered at 250 mg daily for 3 days and 125 mg daily for 3 days, followed by oral prednisolone. The administration of prednisolone was started at 60 mg daily on the 20th day and the prednisolone dose was gradually decreased to 20 mg daily on the 96th day. 

We performed a tracheotomy on the 14th day because we expected that she would need prolonged mechanical ventilation, and she was taken off a ventilator on the 30th day. Chest radiograph and chest CT scan showed a bilateral pulmonary infiltrative shadow that improved on the 64th day and disappeared without lung damage on the 92nd day (Figure 2(f)). 

**Figure 3:**
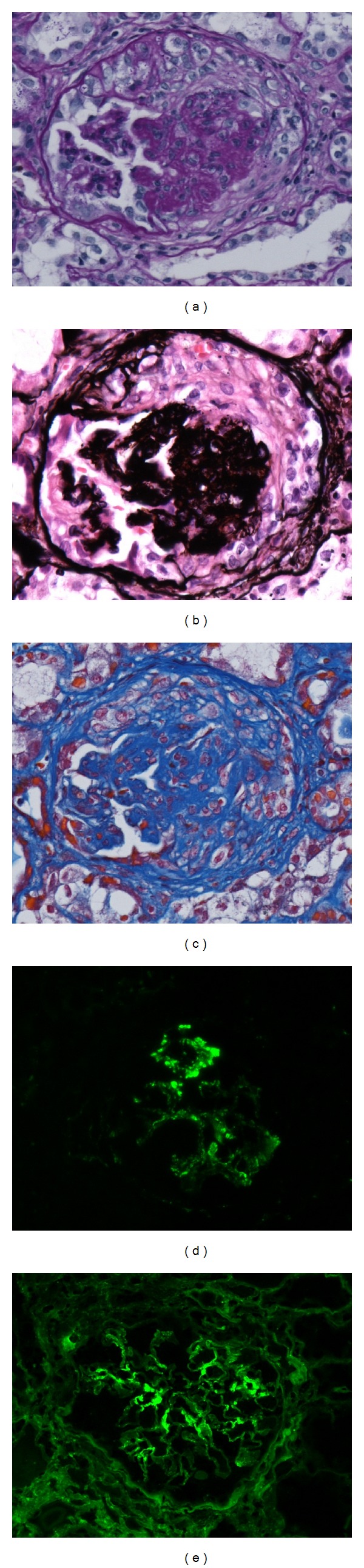
(a), (b), (c) Light-microscopy examination of renal-biopsy specimens showed proliferative-appearing glomeruli with focal formation of cellular and fibrocellular crescents. The GBM was duplicated. Many glomeruli were globally sclerosed, indicating disease chronicity. (A periodic acid Schiff staining, B periodic acid silver-methenamine staining, C Masson's trichrome staining). (d) Immunofluorescence microscopy showed slightly bright granular staining for C3 in the mesangium area. (e) Immunofluorescence microscopy showed weak staining with IgG in a linear pattern in part of the GBM. GBM: glomerular basement membrane.

A kidney biopsy was performed on the 64th day, because her general condition improved and she was able to move to a prone position. The total number of glomeruli was 32, sclerotic glomeruli was 24 (75%), and crescentic glomeruli was 2. The PAS and PAM stain identified sclerotic glomeruli. The linear deposition of IgG was observed in part of the basement membrane by immunofluorescence micrography (see Figure 3). Therefore, although an anti-GBM antibody was not detected in the serum, anti-GBM glomerulonephritis was suspected histopathologically. 

She was transferred to a nearby hospital to continue rehabilitation and maintenance dialysis on the 96th day. One year after onset, there has been no recurrence of PRS with maintenance dosage of prednisolone 10 mg daily.

## 3. Discussion

The term pulmonary renal syndrome (PRS) is used to describe a combination of diffuse pulmonary hemorrhage and glomerulonephritis occurring as the presenting manifestation of multisystem autoimmune disease [3]. The occurrence of pulmonary renal syndrome, which is a rare and etiologically heterogeneous group of diseases, constitutes a medical emergency associated with a high risk of fatal outcome. The outcome data for PRS remain confined to small studies with limited follow-up. Gallagher et al. previously reported 12 of 14 patients who were initially dialysis dependent and 5 patients (36%) who died in the first months in a single-center retrospective study of 14 consecutive patients for four years [4].

On the other hand, the term Goodpasture's syndrome, which has diverse mechanisms and is used synonymously for PRS, is applied to the combination of lung purpura and nephritis, regardless of the underlying pathogenesis. A useful clinical approach to differential diagnosis involves serologic categorization [5].

When evaluating a patient with a possible pulmonary renal syndrome, ANCA and anti-GBM assays currently play a critical role in the diagnosis and classification of vasculitic syndrome. In 1982, antibodies directed against neutrophil cytoplasmic antigens were first described in patients with pauci-immune glomerulonephritis [6].

In Japan, clinical test items for MPO-ANCA antibody and anti-GBM antibody were listed in health-insurance system in 1998 and 1999, respectively.

Single-center experience suggests that 60% to 70% of cases of PRS are associated with autoantibodies to ANCAs and 20% are associated with anti-GBM antibodies [7, 8].

In addition, approximately 95% of the patients with ANCA-associated vasculitis have detectable ANCA, whereas circulating anti-GBM antibodies using ELISAs are found in up to 92% of the cases of anti-GBM disease [9]. That is, approximately 90% of the patients with PRS have one or more autoantibodies. Therefore, uncategorizable PRS with no detection of various antibodies in the serum, as in this case, is extremely rare.

It has been only recently that minimal outcome data for this severe syndrome have become available in the literature. Only five cases of seronegative PRS, of which two reports are written in Japanese and therefore not cited here, have been reported since 1980 [2, 10, 11]. All reported patients are Asian and had already had severe renal dysfunction (BUN: 82 mg/dL–167 mg/dL, Cr: 5.4 mg/dL–11.9 mg/dL) at the time of admission. To preserve organ function, it is important to make the correct diagnosis and institute adequate therapy early on in the acute phase. However, in the present case, differential diagnosis was difficult and better management for PRS could not be performed before she had progressed to end-stage renal failure, as autoantibodies available at our hospital were all negative in multiple examinations.

With regard to therapy for seronegative PRS, no specific therapeutic option that has shown clear benefit has been reported because of the limited numbers of cases. Only two cases that underwent plasma exchange were able to avoid subsequent maintenance dialysis [2].

As it has been described above, although the group of disorders mediated by anti-GBM antibody is one of the less common causes of DAH and nephritis, the prognosis of Goodpasture's syndrome is the worst and the occurrence of anti-GBM antibody with or without other antibodies has been reported to be associated with significantly poor outcome in patients with PRS [12].

In this case, no events to precipitate lung hemorrhage, such as lung infection, were documented and systemic manifestations other than those attributable to uremia or respiratory insufficiency were absent. 

DAH is an acute, life-threatening event, and the causes and clinical features vary widely. Once the diagnosis of diffuse alveolar hemorrhage is established, the clinician should ascertain whether an underlying cause is present.

The diagnosis of DAH was established by dyspnea, cough, hemoptysis, and new alveolar infiltrates in conjugation with bloody bronchoalveolar lavage specimens (with numerous erythrocytes and siderophages). 

The diagnostic evaluation in diffuse alveolar hemorrhage usually includes bronchoscopic examination for two purposes, which are to document alveolar hemorrhage by bronchoalveolar lavage, to exclude airway sources of bleeding by visual inspection and to exclude an associated infection [13].

Although, in the present patient, bronchoscopy was not performed and the lavage specimens were not obtained, DAH was strongly suspected based on the response to steroid therapy, clinical course, the findings of chest CT, and a complication of RPGN.

Although our patient was negative for ANCAs and anti-GBM antibody throughout hospitalization, Salama et al. reported that it is possible to prove the existence of low titer anti-GBM antibody using the Biosensor analysis (biomolecular interaction analysis system) for patients with anti-GBM disease [14]. However, this testing is difficult in the laboratory of our hospital, like many other laboratories, because complicated biochemical methods are required. 

Observing each antibody in greater detail, an antibody titer of P-ANCA was below cutoff value on admission, gradually decreased during hospitalization, and was below the measurement sensitivity on the 119th day. It is not clear whether the decline in antibody titer of P-ANCA within the normal range was a result of a decrease in the disease activity or a good response to treatment with corticosteroids.

In general, when these assays are negative, as in this case, renal biopsy is recommended for all patients with renal involvement because light microscopy and immunofluorescence studies can assess the extent and activity of glomerulonephritis [9]. As the underlying cause remained elusive after a thorough clinical evaluation that included imaging studies and frequent serologic studies, a renal biopsy was performed for diagnostic clues at a later date and specimens were evaluated pathologically. As the specimens showed crescent-shaped changes in the glomeruli, RPGN with crescent formation was diagnosed. However, as most specimens had already progressed to glomerular sclerosis because of the extensive time since the onset, linear IgG deposition along the capillary basement membrane was observed in only a very small number of glomeruli on fluorescent staining. Linear IgG deposition along the capillary basement membrane is virtually diagnostic of Goodpasture's syndrome. Therefore, although accurate diagnosis had been difficult, we thought that the clinical condition of this patient was most likely Goodpasture's syndrome based on clinical and histopathological manifestations.

Glucocorticoid is the main therapy for the DAH syndrome associated with systemic vasculitis, connective tissue disease, and Goodpasture's syndrome. For the treatment of DAH, intravenous pulse methylprednisolone 500 to 2000 mg daily for up to five days was recommended in initial treatment, followed by gradual tapering depending on the response to therapy and then maintenance on an oral preparation [9]. In this patient, 500 mg intravenous methylprednisolone daily for 3 days with a subsequent tapering dose of intravenous methylprednisolone and long-term oral prednisolone were administered.

On the other hand, plasma exchange therapy may also be performed as well as the administration of immunosuppressive agents, such as corticosteroid medications and cyclophosphamide for Goodpasture's syndrome. Plasmapheresis is usually continued for several weeks, until there is a marked decline in serum anti-GBM antibody titers and the patient's hemoptysis stops. In most cases, bleeding in the lungs stops and no permanent lung damage occurs, but damage to the kidneys is long lasting. The merits of plasmapheresis in diffuse alveolar hemorrhage associated with conditions other than Goodpasture's syndrome have not been evaluated in prospective studies [9, 14]. In this case, because we could not establish an accurate diagnosis of Goodpasture's syndrome and we did not detect target autoantibodies to remove through plasmapheresis, we did not perform plasma exchange. In hindsight, as plasma exchange regimens have been shown to be beneficial for patients with uncategorizable PRS, it might have been better to perform plasma exchange, considering that this patient quickly developed end-stage renal failure.

## 4. Conclusion

Uncategorizable pulmonary-renal syndrome, in which double ANCAs and anti-GBM antibodies are negative, is extremely rare and no effective treatment has been established. Since PRS is fatal unless treated promptly, early diagnosis and timely initiation of treatment are crucial for good patient outcome. For these cases, rapid treatment with high-dose corticosteroids coupled with plasma exchange for uncategorizable PRS should be performed, as this was effective in a small subset of patients for preventing end-stage renal disease or death.
